# A virus-derived microRNA-like small RNA serves as a serum biomarker to prioritize the COVID-19 patients at high risk of developing severe disease

**DOI:** 10.1038/s41421-021-00289-8

**Published:** 2021-07-06

**Authors:** Zheng Fu, Jian Wang, Zheng Wang, Ying Sun, Jian Wu, Yongchen Zhang, Xingxiang Liu, Zhen Zhou, Likun Zhou, Chen-Yu Zhang, Yongxiang Yi, Xinyi Xia, Lin Wang, Xi Chen

**Affiliations:** 1 Nanjing Drum Tower Hospital Center of Molecular Diagnostic and Therapy, Chinese Academy of Medical Sciences Research Unit of Extracellular RNA, State Key Laboratory of Pharmaceutical Biotechnology, Jiangsu Engineering Research Center for MicroRNA Biology and Biotechnology, NJU Advanced Institute of Life Sciences (NAILS), Chemistry and Biomedicine Innovation Center, Institute of Artificial Intelligence Biomedicine, School of Life Sciences, Nanjing University, Nanjing, Jiangsu China; 2 Department of Clinical Laboratory, Union Hospital, Tongji Medical College, Huazhong University of Science and Technology, Wuhan, Hubei China; 3 Department of Gastrointestinal Surgery, Union Hospital, Tongji Medical College, Huazhong University of Science and Technology, Wuhan, Hubei China; 4 COVID-19 Research Center, Institute of Laboratory Medicine, Jinling Hospital, Nanjing University School of Medicine, Nanjing, Jiangsu China; 5 Department of Laboratory Medicine, The Second Hospital of Nanjing, Nanjing University of Chinese Medicine, Nanjing, Jiangsu China; 6 Huai’an Fourth Hospital, Huai’an, Jiangsu China; 7 Department of Laboratory Medicine & Blood Transfusion, Wuhan Huoshenshan Hospital, Wuhan, Hubei China; 8 Joint Expert Group for COVID-19, Wuhan Huoshenshan Hospital, Wuhan, Hubei China

**Keywords:** Small RNAs, miRNAs

Dear Editor,

The COVID-19 outbreak has caused a health crisis and economic hardship across the world, and the sudden deterioration of COVID-19 patients into a severe type of illness is a major cause of the high mortality in current pandemic. Since medical facilities do not have reliable biomarkers to predict likelihood of disease progression and identify high-risk patients that require immediate medical attention, patients can only be treated after the appearance of severe symptoms, thereby missing the best treatment window. Furthermore, because patients cannot be stratified at admission, they have to be quarantined and treated without screening, which often leads to high pressure on healthcare services and overwhelming of medical resources. Therefore, it is urgent to develop a biomarker that can accurately predict the severity and prognosis of COVID-19 patients in their pre-severe stage, thereby improving treatment outcome, reducing mortality rate, and assuring proper use of the limited medical resources. Recent studies by us and others have demonstrated that microRNAs (miRNAs), a group of small, single-stranded, noncoding RNAs produced by eukaryotic cells and viruses, circulate in human blood in a highly stable, cell-free form^1,2^. Our further studies have demonstrated that RNA viruses (Ebola^3^ and H5N1^4^) can encode miRNA-like small RNAs (milRNAs) and that circulating viral milRNAs can be exploited for early diagnosis of viral infection and prediction of prognosis^3^. Here, we identified SARS-CoV-2-encoded milRNAs in patients’ sera and evaluated their potential in predicting high-risk individuals before manifestation of severe symptoms.

A total of 159 COVID-19 patients and 51 healthy controls from multiple clinical centers in two provinces of China were enrolled in this study. The baseline characteristics of the COVID‐19 patients are shown in Supplementary Table 1. While there was no significant difference between the severe and mild/moderate patients with respect to sex, there were significant differences in age and comorbidity (Supplementary Fig. S1). A multiphase study was designed to identify SARS-CoV-2-encoded milRNAs and to determine their clinical value in distinguishing severe patients from mild/moderate ones (Fig. 1a; detailed descriptions of the methodology are provided in Supplementary Methods). In screening phase, serum samples were sequenced to identify SARS-CoV-2-encoded milRNAs. In validation phase, serum samples in three cohorts were collected from severe patients who had deteriorated to a severe or critically severe condition or from mild/moderate patients who remained at a mild or moderate condition throughout the hospital stay; milRNAs were absolutely quantified by a quantitative RT-PCR (qRT-PCR) assay to confirm their accuracy in discriminating severe patients from mild/moderate patients. In testing phase, 20 patients with mild or moderate symptoms of COVID‐19 at admission were monitored, and 9 of them showed apparent signs of severe illness during hospitalization and progressed to a severe or critically severe condition, while the other 11 patients remained at a mild or moderate condition throughout their hospital stay; milRNAs were monitored across different time points to examine whether they could predict disease severity ahead of manifestation of severe symptoms.

**Fig. 1:**
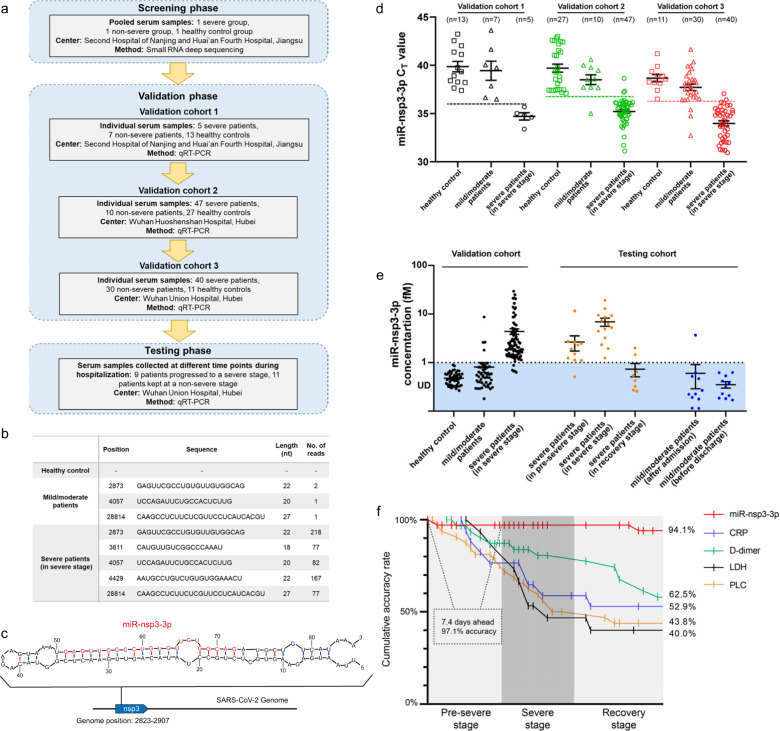
Identification and detection of milRNAs of SARS-CoV-2 in the sera from COVID-19 patients. **a** Overview of the study design. **b** SARS-CoV-2-derived small RNAs in the sera of COVID-19 patients and healthy controls. **c** Genomic position and stem-loop structure of the precursor of miR-nsp3-3p. Mature miR-nsp3-3p is indicated in red. **d** The individual *C*_*T*_ values of miR-nsp3-3p in the serum from severe patients, mild/moderate patients, and healthy controls in the validation cohorts 1, 2, and 3. Serum samples were collected from mild/moderate patients who remained at a mild or moderate condition or from severe patients who had developed a severe or critically severe illness. The lower boundary of the detection spectrum of each cohort is indicated by a dotted line. **e** The absolute concentrations of miR-nsp3-3p in the serum from validation and testing cohorts. In testing cohort, serum samples were collected from mild/moderate patients at the time of admission and before discharge or from severe patients when the disease progressed from pre-severe stage (before manifestation of severe symptoms) to severe stage and then to recovery stage. **f** The cumulative accuracy rate of miR-nsp3-3p, D-dimer, CRP, LDH, and PLC in predicting the risk of critical illness and monitoring disease progression.

In screening phase, abundant endogenous miRNAs were detected in the sera from severe or mild/moderate patients and healthy controls by small RNA deep sequencing (Supplementary Fig. S2a). While no small RNA (18–30 nucleotides) from SARS-CoV-2 was detected in healthy controls and only 4 sequencing reads (corresponding to three viral small RNAs) were detected in mild/moderate patients, 621 reads (corresponding to five viral small RNAs) were detected in severe patients (Fig. 1b). Since miRNA precursor usually folds into a stem-loop hairpin structure and mature miRNA is usually located on a hairpin arm, the SARS-CoV-2 genome was scanned for stem-loop structures that comprised the small RNAs on hairpin arm. Only one small RNA fulfilled the criteria and could be classified as a milRNA. This milRNA was located at nucleotides 2873–2894, on the 3′ arm of a hairpin in the nsp3 gene (hereafter, miR-nsp3-3p) (Fig. 1c). MiR-nsp3-3p was conserved among SARS-CoV-2 strains and had at least three mismatches to other Coronaviruses and other types of viruses (Supplementary Fig. S2b).

Next, miR-nsp3-3p was refined by qRT-PCR in three validation cohorts. The dynamic range and sensitivity of the qRT-PCR assay for measuring miR-nsp3-3p was first determined. The lower boundary of the detection spectrum was 0.01 attomole, corresponding to the cutoff *C*_*T*_ values of 35.90, 36.50, and 36.40 in validation cohort 1, 2, and 3, respectively (Supplementary Fig. S2c). According to the cutoff *C*_*T*_ values, the *C*_*T*_ values were consistently within the detection range for severe patients but outside for mild/moderate patients and healthy controls (Fig. 1d). Out of 139 patients and 51 healthy controls, only 5 severe patients and 4 mild/moderate patients were misclassified. By referring to the standard curve, the absolute concentrations of miR-nsp3-3p ranged from 1.05 to 29.40 fM in severe patients’ sera (Fig. 1e). When comparing to D-dimer, C-reactive protein (CRP), lactate dehydrogenases (LDH), and peripheral lymphocyte count (PLC), whose levels changed significantly in severe patients (Supplementary Fig. S2e–h), miR-nsp3-3p was superior to these conventional biochemical characteristics for stratification of severe patients from mild/moderate ones and had a larger AUC (0.933) than D-dimer (0.716), CRP (0.788), LDH (0.743), and PLC (0.835) in ROC curve analysis (Supplementary Fig. S2d).

In fact, RNA viruses are not expected to encode miRNAs, because the miRNAs themselves will target the viral genome and inhibit the viral replication. However, recent studies have revealed that some negative- (e.g., H5N1 influenza and Ebola viruses) and positive-stranded (e.g., West Nile and Dengue viruses) RNA viruses are capable of expressing milRNAs^5^. To confirm the presence of milRNAs in COVID-19 patients, SARS-CoV-2 NSP3 and N genes comprising the five viral small RNAs selected by deep sequencing were cloned into a pcDNA6/myc-His B plasmid and transfected into HEK293T cells, respectively. Although these five small RNAs were successfully generated in transfected cells (Supplementary Fig. S3a), only miR-nsp3-3p could be detected in exosomes from cell culture supernatants (Supplementary Fig. S3b). To confirm that the stem-loop structure is indispensable for the maturation of miR-nsp3-3p, wild-type or mutant precursor of miR-nsp3-3p was cloned into a plasmid and transfected into HEK293T cells. While wild-type precursor could be smoothly processed into mature miR-nsp3-3p in cellular environment, a mutation introduced into the stem-loop structure abolished the detection of miR-nsp3-3p in transfected cells (Supplementary Fig. S3c). Consistent with the results from in vitro assay, qRT-PCR analysis of the five small RNAs followed by TA-cloning and sequencing of the PCR products confirmed correct amplification of miR-nsp3-3p in severe patients’ sera (Supplementary Fig. S3d). In contrast, qRT-PCR analysis of the other four viral small RNAs in the sera of severe patients and healthy controls showed no significant difference, suggesting absence of substantial viral small RNAs in patients’ sera (Supplementary Fig. S3e). These results indicate that the biogenesis and biodistribution of miR-nsp3-3p are different from the rest viral small RNAs. According to previous reports^4,6^, SARS-CoV-2 may employ RNA endonucleases (e.g., Argonaute 2) to cut the stem-loop structure of miR-nsp3-3p in cytoplasm and exploit the secretory exosomes to spread miR-nsp3-3p to blood circulation.

Finally, miR-nsp3-3p was monitored along with the 20 patients of testing cohort who had undergone follow-up exams. For the 9 patients progressing from mild/moderate to severe symptoms, positive miR-nsp3-3p signals were consistently detected in their sera at the pre-severe stage; then the signals remained positive at the severe stage; until recovery stage, miR-nsp3-3p dropped to undetectable levels (Fig. 1e). In contrast, PLC, LDH, CRP, and D-dimer usually showed a normal distribution at pre-severe stage, and only in a portion of cases they were abnormally altered at severe stage and could be recovered to normal levels at recovery stage (Supplementary Fig. S4). The prediction horizon and accuracy of each index in predicting the risk of critical illness and evaluating disease progression were further analyzed. On average, miR-nsp3-3p could predict the severe type of disease 7.4 days in advance of severe symptoms with an accuracy of 97.1% (Fig. 1f). Furthermore, miR-nsp3-3p could accurately monitor the trend of disease progression during patients’ hospital stay, with a cumulative accuracy of 94.1%, which was much higher than the other four indices (Fig. 1f). For the other 11 patients remaining at a mild or moderate condition, only miR-nsp3-3p and PLC showed a normal distribution both at the time of admission and throughout the hospital stay, whereas D-dimer, LDH, and CRP were often misclassified as abnormal in these patients (Supplementary Fig. S5). Overall, miR-nsp3-3p is able to capture the risk of critical illness far ahead of the clinical outcome and is superior over other indices for evaluating the disease development and recovery in COVID-19 patients.

The ability to precisely prioritize the patients that are at a high risk of developing severe disease is vital. At present, it is possible to predict COVID-19 prognosis using symptoms, signs, radiographic abnormalities, and clinical parameters^7^, but these features are often seen in the late course of disease progression and highly variable from region to region and country to country. On the other hand, although some researches have shown that transcriptomics, proteomics, or metabolomics analysis of COVID-19 patients could be used to precisely characterize severity status, these techniques are too complex for rapid and general application in the current pandemic. In this study, we showed that miR-nsp3-3p was superior to D-dimer, CRP, LDH, and PLC for stratification of severe patients from mild/moderate ones and could identify high-risk individuals in advance of the manifestation of severe symptoms. Because miR-nsp3-3p exhibits comparable sensitivity, specificity, and precision to that of multi-omics, and because the quantification protocol is quite simple, this new biomarker can be readily applied in the current COVID-19 pandemic and provide a simple and operable decision tool to prioritize high-risk patients, especially in the places with shortages of medical resources, thereby allowing more effective control of the pandemic and relief of social economic burdens.

## Supplementary information

Supplementary Figures and Tables
