# Supplementary material for: A virus-derived microRNA-like small RNA serves as a serum biomarker to prioritize the COVID-19 patients at high risk of developing severe disease
Source: Cell Discov. 2021 Jul 6;7:48. doi: 10.1038/s41421-021-00289-8 (PMC8257610; doi:10.1038/s41421-021-00289-8)
Supplement: Supplementary file 1 — Supplementary Figures and Tables [file 41421_2021_289_MOESM1_ESM.pdf]

# Supplementary information for
# A virus-derived microRNA-like small RNA serves as a serum biomarker to prioritize the COVID-19 patients at high risk of developing severe disease

**(Zheng Fu *et al.*)**

**Supplementary Methods**

**Enrolment of patients**

According to the COVID-19 Diagnosis and Treatment Guideline issued by the National Health Committee of China (7th Edition) [1] and the World Health Organization interim guidance [2], patients were diagnosed by the throat swab and RT-PCR method and were classified into four types: mild, moderate, severe, and critically severe. Mild: mild clinical symptoms and no radiological changes. Moderate: fever, respiratory distress and CT scans indicating pneumonia signs. Severe: meeting any of the followings: respiratory distress (respiratory rate $\geq$ 30 times per min); oxygen saturation $\leq$ 93% in the resting state; partial pressure of arterial blood oxygen ($PaO_2$)/fraction of inspired oxygen ($FiO_2$) $\leq$ 300 mmHg; and CT chest imaging showing that lung damage developed significantly within 24 to 48 h. Critically severe: meeting any of the followings: respiratory failure requiring mechanical ventilation; signs of septic shock; and multiple organ failure requiring ICU admission.

A total of 159 COVID-19 patients and 51 healthy controls from multiple clinical centres in two provinces of China were enrolled in this study. Samples in the screening cohort/validation cohort 1 were collected at the Second Hospital of Nanjing (Nanjing, Jiangsu, China) and Huai'an Fourth Hospital (Huai'an, Jiangsu, China); samples in the validation cohort 2 were collected at Wuhan Huoshenshan Hospital (Wuhan, Hubei, China); samples in the validation cohort 3 were collected at Wuhan Union Hospital (Wuhan, Hubei, China); and samples in the testing cohort were collected at Wuhan Union Hospital. Patient's demographic (age, gender, severity of illness and comorbidity conditions) and clinical (D-dimer, CRP, LDH and PLC) data were collected from each centre. Of the 159 patients enrolled, the average age was 63.4 years (ranging from 24 to 97 years), 91 (56.8%) were male, and 93 patients (58.5%) had one or more comorbidities (hypertension, diabetes, cardiovascular diseases, chronic respiratory diseases, etc.).

For the validation cohorts, patients who deteriorated to a severe or critically severe condition were assigned to the severe group, and patients who remained at a mild or moderate state were assigned to the mild/moderate group; the levels of miR-nsp3-3p, D-dimer, CRP, LDH and PLC were quantified to evaluate their accuracy in discriminating severe patients from mild/moderate ones. For the testing cohort, a prospective analysis was conducted on 20 patients who had undergone follow-up exams after hospital admission. Nine patients displayed only mild or moderate respiratory symptoms (e.g., fever and coughing) at admission, but they showed apparent signs of severe illness (respiratory distress and less than 93% of oxygen saturation) during hospitalization and progressed to a severe or critically severe phase. After treatment, 8 patients moved towards the recovery phase and were finally discharged from the hospital, whereas 1 patient died. The other 11 patients experienced only mild or moderate symptoms throughout their hospital stay. Most patients had multiple blood samples taken during the observational period, and the levels of miR-nsp3-3p, D-dimer, CRP, LDH and PLC were quantified to calculate the risk of developing severe illness at hospital admission or at follow-up exam times. The study protocol was approved by the Ethics Committee of each participating hospital. Written informed consent was waived in view of the new emerging infectious diseases in a designated hospital. Blood samples were heat-inactivated at 56°C for 30 min, and then the serum was isolated and stored at -20°C before use.

**Small RNA deep sequencing and computational analysis of viral milRNAs**

For small RNA deep sequencing, serum samples from the screening cohort were pooled from severe patients, mild/moderate patients and healthy controls, respectively (each pooled from 3-5 individuals). Equal volume of pooled serum samples (1 mL) was subjected to RNA extraction with TRIzol reagent according to the manufacturer's instructions. Small RNA deep sequencing of serum RNA samples was performed by BGI (Shenzhen, China). Reference genomic sequences of SARS-CoV-2 (NC_045512) were downloaded from the NCBI database. After removing the adaptor sequences from the raw data, clean reads were compared to the known human miRNA precursors in the miRBase database 22.1 based on the Smith-Waterman algorithm. Only candidates with no mismatches and no more than 2 shifts were counted as endogenous miRNAs. For analysis of viral milRNAs, clean reads were aligned against the SARS-CoV-2 full genome to search for virus-derived small RNAs. Then the sequences of viral small RNAs were excised twice, once including 70 nt upstream and 20 nt downstream flanking sequence, and once including 20 nt upstream and 70 nt downstream flanking sequence. Mfold was used to predict the RNA secondary structures of the excised sequences. Only when the excised sequence folded into a stem-loop hairpin structure and the viral small RNA was located on the 5' or 3' arm of the hairpin, this virus-derived small RNA could be counted as a milRNA. To confirm the specificity of the SARS-CoV-2 milRNAs, we used bowtie to scan full-length genomes of SARS-CoV-2 strains retrieved from GISAID (www.gisaid.org) and other viral genomes including Coronaviruses retrieved from NCBI genome database.

**qRT-PCR assay for viral milRNAs**

Total RNA was extracted from 200 µL of serum with TRIzol reagent according to the manufacturer's instructions. The qRT-PCR procedure for miR-nsp3-3p was performed using a Vazyme miRNA PCR Kit (Vazyme, Nanjing, China) according to the manufacturer's instructions. Briefly, 2 µL of total RNA was reverse-transcribed to cDNA using the miRNA 1st Strand cDNA Synthesis Kit (by stem-loop) and stem-loop RT primers (miR-nsp3-3p-rt: 5'-GTCGTATCCAGTGCAGGGTCCGAGGTATTCGCACTGGATACGACCTGCCA-3'). The reaction conditions were as follows: 25°C for 5 min, 50°C for 15 min and 85°C for 5 min. The product was further amplified with real-time PCR, which was performed with specific primers (miR-nsp3-3p-f: 5'-GCGGAGTTCGCCTGTGTTG-3') and universal reverse primer (5'-AGTGCAGGGTCCGAGGTATT-3') using Vazyme miRNA Universal SYBR qPCR Master Mix (Vazyme) and a Roche LightCycler 96 (Roche). The reactions were incubated in a 96-well optical plate at 95°C for 5 min, followed by 40 cycles of 95°C for 10 sec and 60°C for 30 sec. After the reactions, the cycle threshold ($C_T$) values were determined using fixed threshold settings, and the mean $C_T$ values were determined from triplicate PCRs. For absolute quantification, a series of synthetic miR-nsp3-3p at diluted concentrations was reverse-transcribed and amplified, and a no-template control was assessed simultaneously to determine the specificity of the primer set. The absolute expression levels of miR-nsp3-3p were then calculated according to the standard curve.

**Plasmid construction**

Mammalian expression plasmids (pcDNA6.2-GW-miR) encoding wild-type miR-nsp3-3p precursor and its mutant form (miR-nsp3-3p precursor$^{mut}$) were purchased from Genescript (Genescript, Nanjing, China). Sequence in miR-nsp3-3p precursor was mutated (from GAGTTCGCCTGTGTTGTGGCAGATGCTGTCATAAA to GAGTTCGCCTGTGTTGTGGCAGAGTAGGGACTAAA). An empty plasmid served as a

negative control. Mammalian expression plasmid encoding SARS-CoV-2 NSP3 gene and N gene were generously provided by Prof. Peihui Wang (Shandong University, Jinan, China). The expression plasmids were transfected into HEK293T cells using Lipofectamine 2000 (Invitrogen) according to the manufacturer's instructions. Total RNA was isolated at 24 h post-transfection.

**Statistical analysis**

All of the data from the qRT-PCR analyses were obtained from at least three independent experiments. The data shown are presented as the means ± SEM. Statistical analysis was performed with Graphpad 8.0. Two-sided $\chi 2$ test was used to compare the differences in variables among the groups. Normal distribution of data was assessed in all cases by Shapiro–Wilk test. Data following or not following a normal distribution were analysed with parametric (one-way analysis of variance (ANOVA) with Dunnett's multiple comparison post-test) or non-parametric (Kruskal–Wallis ANOVA with Dunn's multiple comparison post-test) statistics, respectively. Differences were considered significant at $P < 0.05$.

**References**


1. NHC. New corona virus pneumonia prevention and control program (7th edition). (http://www.nhc.gov.cn/yzygj/s7653p/202003/46c9294a7dfe4cef80dc7f5912eb1989/files/ce3e6945832a438eaae415350a8ce964.pdf, 2020).
2. WHO. Clinical management of COVID-19. (https://www.who.int/publications-detail/clinical-management-of-covid-19, 2020).

## Supplementary Figures

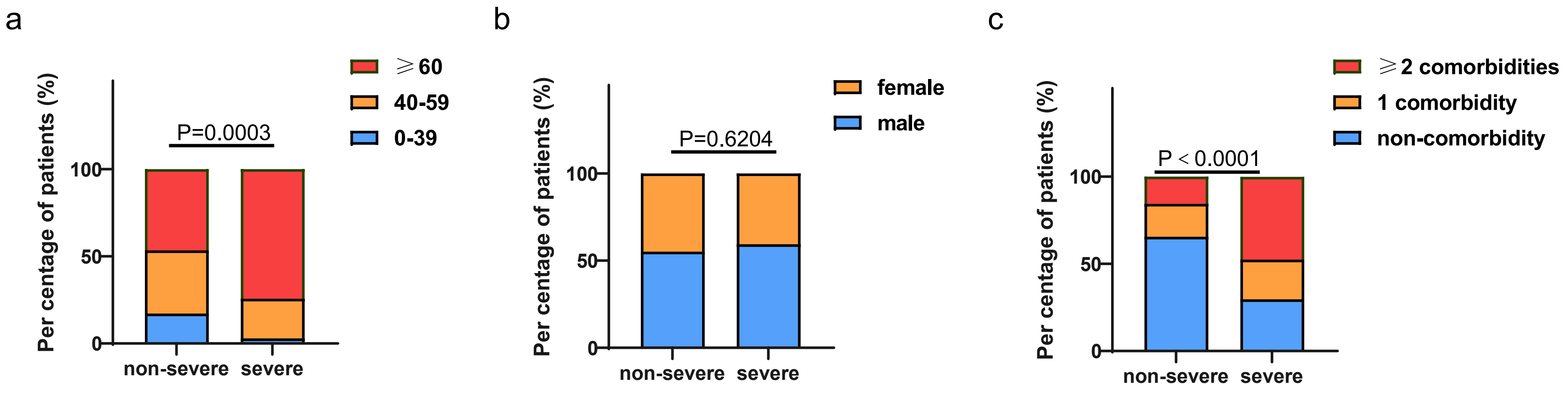


**Figure S1. Analyses of demographic parameters of enrolled COVID-19 patients. (a)** Bar graph showing the percentages of male and female patients. Two-sided χ2 test. **(b)** Bar graph showing the percentages of patients in high, middle and low age groups. Two-sided χ2 test. **(c)** Bar graph showing the percentages of patients without or with 1 or more comorbidities. Two-sided χ2 test.

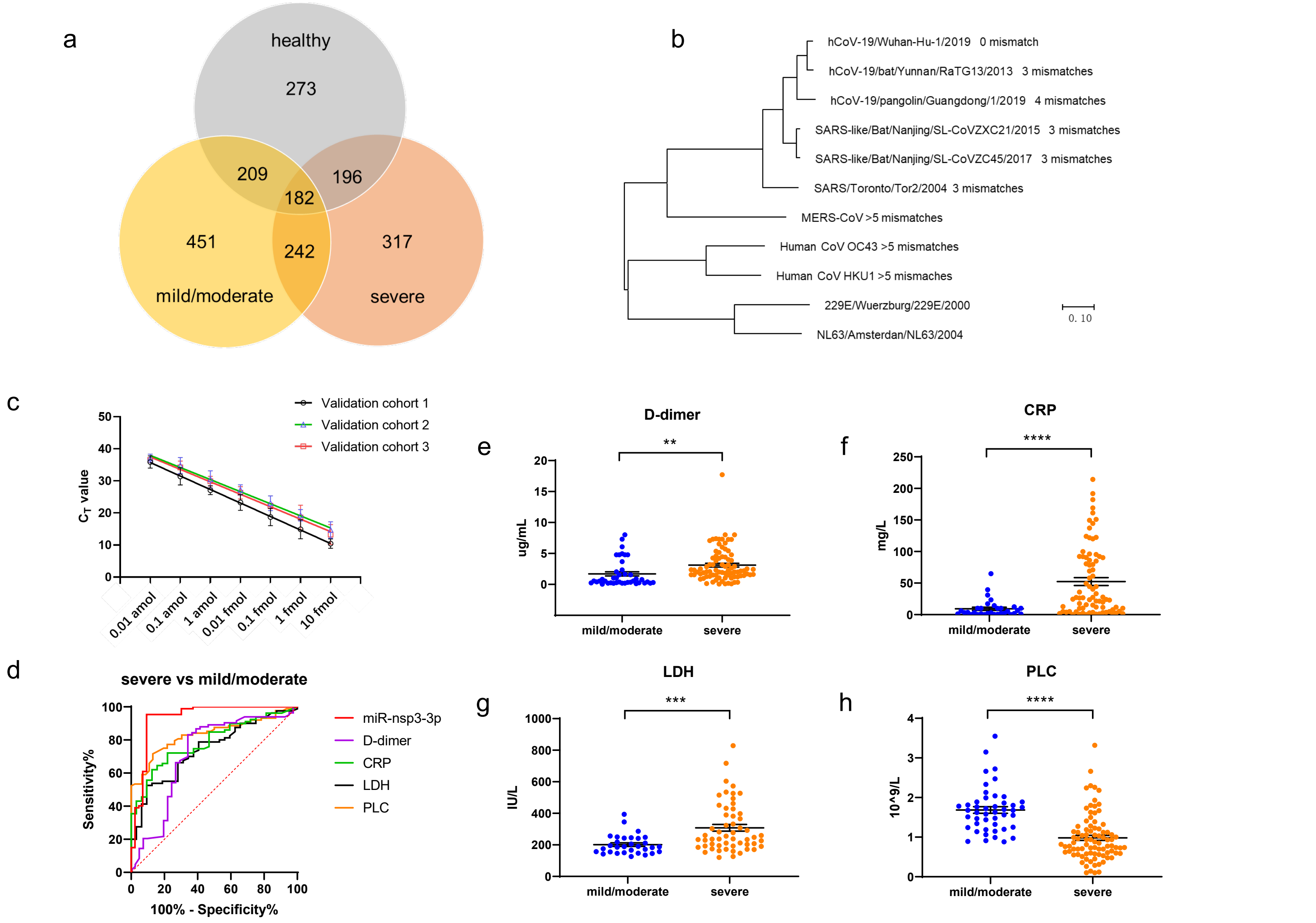


**Figure S2. Detection of endogenous miRNAs, viral milRNA and other indexes in the sera from COVID-19 patients. (a)** Number of overlapped endogenous miRNAs in the sera of severe or mild/moderate patients and healthy controls. **(b)** MiR-nsp3-3p was conserved in SARS-CoV-2 strains but not in other Coronaviruses and other types of viruses. **(c)** The dynamic range and sensitivity of the qRT-PCR assay for measuring miR-nsp3-3p. Synthetic single-stranded miR-nsp3-3p was serially diluted over several orders of magnitude, corresponding to levels ranging from 0.01 attomole to 10 femtomole and was assessed via qRT-PCR. The resulting $C_T$ values were plotted against the amount of input miR-nsp3-3p to generate a standard curve. Water was used in place of RNA as a no-template control (background) for the qRT-PCR assay. According to the lower boundary of the detection spectrum, $C_T$ values of 35.90, 37.05 and 36.40 were set as the cut-off points for validation cohort 1, 2 and 3, respectively. **(d)** ROC curve analysis for discrimination between severe and mild/moderate patients by miR-nsp3-3p, D-dimer, CRP, LDH and PLC levels in the validation cohort. **(e-h)** The levels of D-dimer, CRP, LDH and PLC were compared between mild/moderate and severe patients in the validation cohort.

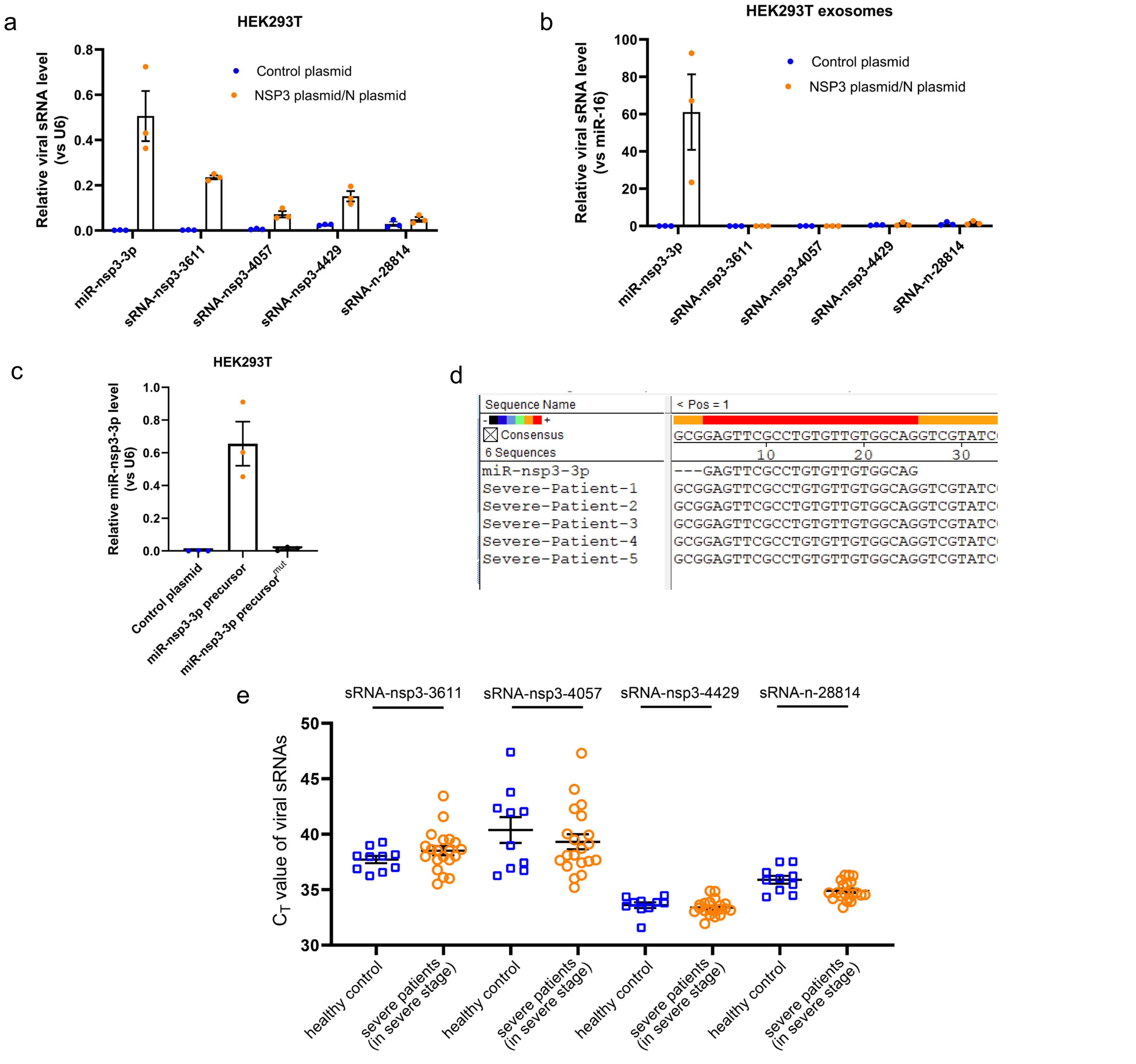


**Figure S3.** ***In vitro* characterization of viral small RNAs (sRNAs). (a)** The relative expression level of viral sRNAs in HEK293T cells transfected with plasmids that express SARS-CoV-2 NSP3 or N gene. An empty plasmid was used as a negative control. **(b)** Quantitative RT-PCR analysis of viral sRNAs in exosomes derived from HEK293T cells that were transfected with the plasmids that express SARS-CoV-2 NSP3 or N gene. **(c)** The relative expression level of mature miR-nsp3-3p in HEK293T cells that were transfected with a pcDNA6.2-GW-miR plasmid expressing miR-nsp3-3p precursor sequence. An empty plasmid or mutant miR-nsp3-3p precursor was used as a negative control. **(d)** The alignment result of TA-cloning and sequencing of qRT-PCR products. RNA isolated from sera of severe patients was subjected to qRT-PCR, and then the product was ligated into a TA-vector and sequenced. **(e)** The $C_T$ values of 4 viral sRNAs in the sera from 20 severe patients and 10 healthy controls (randomly picked from validation cohort 2).

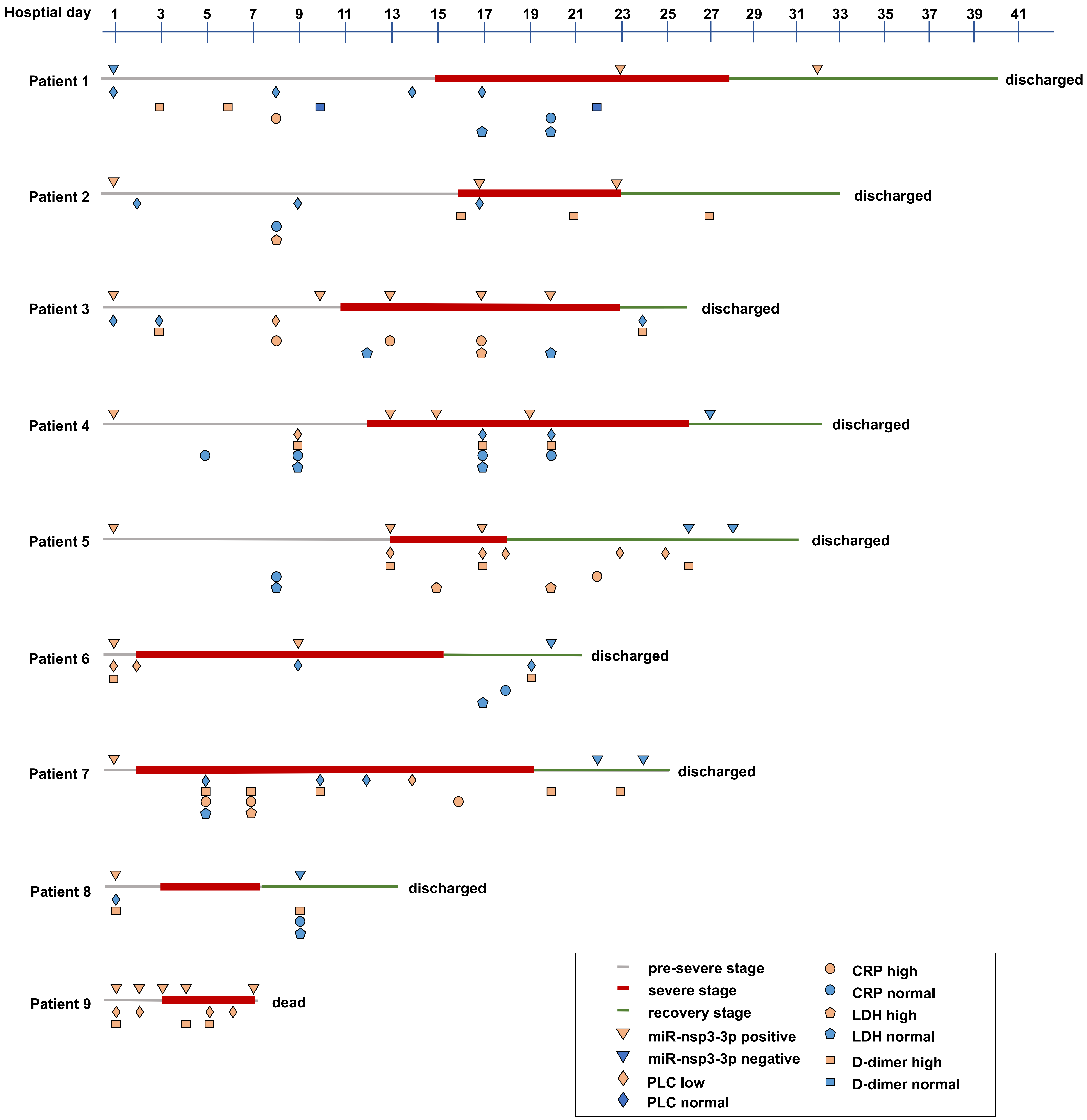


**Figure S4. Diagram of detection of miR-nsp3-3p, D-dimer, CRP, LDH and PLC in patients who progressed to a severe/critically severe stage.** X-axis means the hospital day of patients and the Y-axis means different indexes. Day 1 represents the day of hospitalization. Grey lines indicate the pre-severe stage, red lines indicate the severe stage, and green lines indicate the recovery stage. Each triangle, rectangle, circle, pentagon or diamond marker indicates a follow-up exam of miRnsp3-3p, D-dimer, CRP, LDH or PLC, respectively.

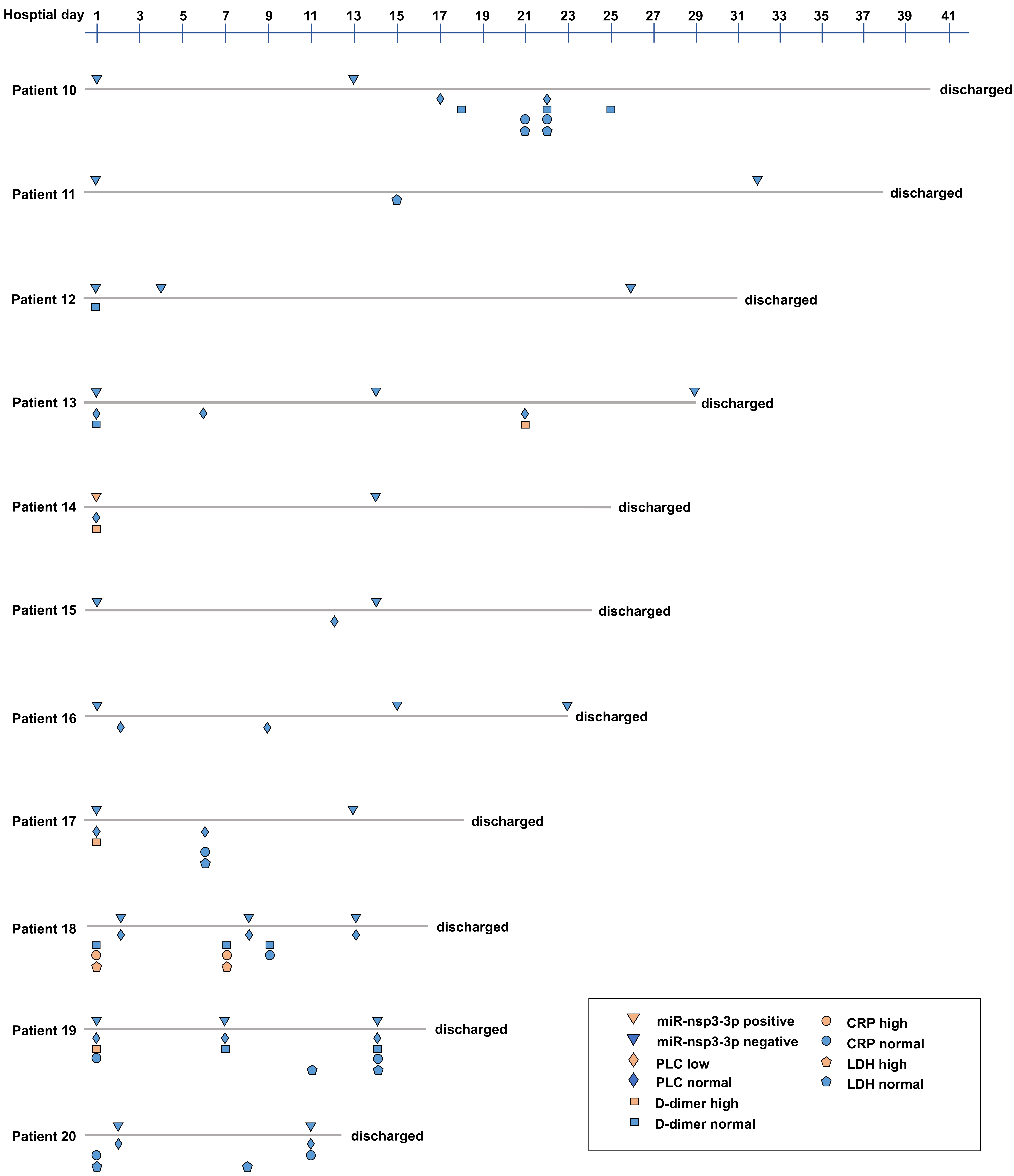


**Figure S5. Diagram of detection of miR-nsp3-3p, D-dimer, CRP, LDH and PLC in patients who remained at a mild/moderate stage.** X-axis means the hospital day of patients and the Y-axis means different indexes. Day 1 represents the day of hospitalization. Each triangle, rectangle, circle, pentagon or diamond marker indicates a follow-up exam of miR-nsp3-3p, D-dimer, CRP, LDH or PLC, respectively.

## Supplementary tables

## Table S1. Demographic and clinical features of the COVID-19 patients and normal controls.

| | Screening cohort/ Validation cohort 1 | | | Validation cohort 2 | | | Validation cohort 3 | | | Testing cohort | |
|---|---|---|---|---|---|---|---|---|---|---|---|
| | Healthy control | Non-severe patient | Severe patient | Healthy control | Non-severe patient | Severe patient | Healthy control | Non-severe patient | Severe patient | Non-severe patient | Severe patient |
| **Age group** | | | | | | | | | | | |
| **0-19** | | | | | | | | | | | |
| **20-39** | 3 | 3 | | 2 | | | 9 | 4 | 1 | 3 | 2 |
| **40-59** | 5 | 4 | 1 | 7 | 6 | 7 | 2 | 7 | 13 | 4 | 2 |
| **≥60** | 5 | | 4 | 18 | 4 | 40 | | 19 | 26 | 4 | 5 |
| **Gender** | | | | | | | | | | | |
| **Male** | 4 | 2 | 2 | 10 | 6 | 30 | 5 | 17 | 22 | 7 | 6 |
| **Female** | 9 | 5 | 3 | 17 | 4 | 17 | 6 | 13 | 18 | 4 | 3 |
| **Severity of illness** | | | | | | | | | | | |
| **Mild** | | | | | | | | 6 | | 1 | |
| **Moderate** | | 7 | | | 10 | | | 24 | | 10 | |
| **Severe** | | | 4 | | | 24 | | | 19 | | 5 |
| **Critically severe** | | | 1 | | | 23 | | | 21 | | 4 |
| **Comorbid conditions** | | | | | | | | | | | |
| **Non-comorbidity** | | 5 | | | 6 | 11 | | 17 | 15 | 10 | 4 |
| **1 comorbidity** | | 2 | 2 | | 3 | 7 | | 5 | 13 | 1 | 1 |
| **≥2 comorbidities** | | | 3 | | 1 | 29 | | 8 | 12 | | 4 |
| **Total** | **13** | **7** | **5** | **27** | **10** | **47** | **11** | **30** | **40** | **11** | **9** |

**Table S2. CT values of miR-nsp3-3p and SARS-CoV-2 genomic RNA.**

**Screening cohort/Validation cohort**

| Screening cohort/Validation cohort 1 | miR-nsp3-3p $C_T$ value | $C_T$ value of viral genome |
|---|---|---|
| Mild/Moderate Patient 1 | 43.63 | 39.08 |
| Mild/Moderate Patient 2 | 37.85 | 23.59 |
| Mild/Moderate Patient 3 | 41.64 | 41.47 |
| Mild/Moderate Patient 4 | 39.73 | 35.08 |
| Mild/Moderate Patient 5 | 36.65 | 34.73 |
| Mild/Moderate Patient 6 | 36.49 | 32.37 |
| Mild/Moderate Patient 7 | 40.03 | 31.78 |
| Severe Patient 1 | 34.60 | 31.84 |
| Severe Patient 2 | 33.38 | 30.74 |
| Severe Patient 3 | 35.68 | 25.70 |
| Severe Patient 4 | 34.83 | 24.92 |
| Severe Patient 5 | 35.02 | 30.11 |

| Validation cohort 2 | miR-nsp3-3p $C_T$ value | $C_T$ value of viral genome |
|---|---|---|
| Mild/Moderate Patient 1 | 35.01 | 28.12 |
| Mild/Moderate Patient 2 | 39.45 | 26.07 |
| Mild/Moderate Patient 3 | 40.58 | 27.71 |
| Mild/Moderate Patient 4 | 39.77 | 27.25 |
| Mild/Moderate Patient 5 | 40.01 | 27.68 |
| Mild/Moderate Patient 6 | 37.72 | 28.61 |
| Mild/Moderate Patient 7 | 38.51 | 31.20 |
| Mild/Moderate Patient 8 | 37.69 | 29.68 |
| Mild/Moderate Patient 9 | 38.58 | 30.47 |
| Mild/Moderate Patient 10 | 37.72 | 25.38 |
| Severe Patient 1 | 34.76 | 27.07 |
| Severe Patient 2 | 35.91 | 32.02 |
| Severe Patient 3 | 35.68 | 28.76 |
| Severe Patient 4 | 35.39 | 27.07 |
| Severe Patient 5 | 31.10 | 28.26 |
| Severe Patient 6 | 36.31 | 27.34 |
| Severe Patient 7 | 36.07 | 27.26 |
| Severe Patient 8 | 35.02 | 28.45 |
| Severe Patient 9 | 34.89 | 31.51 |
| Severe Patient 10 | 34.78 | 22.46 |
| Severe Patient 11 | 36.13 | 26.27 |
| Severe Patient 12 | 35.68 | 29.36 |
| Severe Patient 13 | 36.09 | 24.59 |
| Severe Patient 14 | 36.01 | 30.03 |
| Severe Patient 15 | 34.94 | 32.62 |
| Severe Patient 16 | 35.96 | 27.41 |
| Severe Patient 17 | 36.17 | 24.31 |
| Severe Patient 18 | 37.31 | 27.65 |
| Severe Patient 19 | 36.18 | 33.93 |
| Severe Patient 20 | 35.39 | 31.76 |
| Severe Patient 21 | 35.63 | 27.38 |
| Severe Patient 22 | 35.67 | 30.25 |
| Severe Patient 23 | 34.38 | 28.80 |

| | | |
|---|---|---|
| Severe Patient 24 | 33.94 | 34.48 |
| Severe Patient 25 | 37.22 | 28.68 |
| Severe Patient 26 | 34.93 | 29.36 |
| Severe Patient 27 | 36.20 | 30.91 |
| Severe Patient 28 | 34.66 | 26.90 |
| Severe Patient 29 | 35.74 | 25.41 |
| Severe Patient 30 | 35.30 | 23.22 |
| Severe Patient 31 | 36.04 | 26.32 |
| Severe Patient 32 | 35.21 | 34.12 |
| Severe Patient 33 | 34.87 | 22.71 |
| Severe Patient 34 | 33.68 | 27.71 |
| Severe Patient 35 | 35.54 | 28.83 |
| Severe Patient 36 | 35.89 | 25.86 |
| Severe Patient 37 | 38.64 | 25.63 |
| Severe Patient 38 | 34.12 | 23.74 |
| Severe Patient 39 | 36.14 | 28.67 |
| Severe Patient 40 | 34.19 | 28.27 |
| Severe Patient 41 | 33.51 | 25.05 |
| Severe Patient 42 | 31.69 | 26.27 |
| Severe Patient 43 | 32.55 | 26.36 |
| Severe Patient 44 | 33.73 | 30.35 |
| Severe Patient 45 | 34.40 | 24.97 |
| Severe Patient 46 | 36.35 | 33.24 |
| Severe Patient 47 | 34.79 | 30.95 |

| **Validation cohort 3** | **miR-nsp3-3p $C_T$ value** | **$C_T$ value of viral genome** |
|---|---|---|
| Mild/Moderate Patient 1 | 36.46 | 35.85 |
| Mild/Moderate Patient 2 | 39.64 | 35.60 |
| Mild/Moderate Patient 3 | 41.65 | 38.40 |
| Mild/Moderate Patient 4 | 34.75 | 33.41 |
| Mild/Moderate Patient 5 | 38.13 | 36.95 |
| Mild/Moderate Patient 6 | 39.90 | 35.19 |
| Mild/Moderate Patient 7 | 36.95 | 25.63 |
| Mild/Moderate Patient 8 | 37.79 | 24.57 |
| Mild/Moderate Patient 9 | 36.74 | 40.56 |
| Mild/Moderate Patient 10 | 38.90 | 31.16 |
| Mild/Moderate Patient 11 | 39.91 | 38.27 |
| Mild/Moderate Patient 12 | 36.55 | 32.69 |
| Mild/Moderate Patient 13 | 38.07 | 36.81 |
| Mild/Moderate Patient 14 | 38.46 | 36.10 |
| Mild/Moderate Patient 15 | 38.49 | 30.82 |
| Mild/Moderate Patient 16 | 40.35 | 36.77 |
| Mild/Moderate Patient 17 | 35.38 | 39.47 |
| Mild/Moderate Patient 18 | 37.24 | 34.12 |
| Mild/Moderate Patient 19 | 38.56 | 36.42 |
| Mild/Moderate Patient 20 | 37.14 | 34.19 |
| Mild/Moderate Patient 21 | 37.56 | 35.84 |
| Mild/Moderate Patient 22 | 36.43 | 34.80 |
| Mild/Moderate Patient 23 | 38.36 | 37.88 |
| Mild/Moderate Patient 24 | 39.28 | 31.22 |
| Mild/Moderate Patient 25 | 38.20 | 37.09 |
| Mild/Moderate Patient 26 | 32.72 | 35.87 |
| Mild/Moderate Patient 27 | 36.42 | 32.91 |

| | | |
|---|---|---|
| Mild/Moderate Patient 28 | 36.69 | 35.94 |
| Mild/Moderate Patient 29 | 37.14 | 32.77 |
| Mild/Moderate Patient 30 | 37.53 | 36.71 |
| Severe Patient 1 | 34.29 | 26.68 |
| Severe Patient 2 | 36.17 | 24.36 |
| Severe Patient 3 | 35.01 | 37.39 |
| Severe Patient 4 | 31.21 | 37.46 |
| Severe Patient 5 | 32.06 | 35.80 |
| Severe Patient 6 | 32.73 | 37.04 |
| Severe Patient 7 | 34.73 | 25.49 |
| Severe Patient 8 | 33.66 | 33.06 |
| Severe Patient 9 | 34.52 | 27.35 |
| Severe Patient 10 | 33.67 | 27.56 |
| Severe Patient 11 | 35.71 | 33.98 |
| Severe Patient 12 | 34.82 | 28.48 |
| Severe Patient 13 | 32.69 | 30.90 |
| Severe Patient 14 | 33.86 | 36.92 |
| Severe Patient 15 | 30.92 | 33.72 |
| Severe Patient 16 | 34.82 | 37.87 |
| Severe Patient 17 | 36.42 | 35.38 |
| Severe Patient 18 | 33.96 | 33.78 |
| Severe Patient 19 | 31.97 | 28.56 |
| Severe Patient 20 | 32.00 | 30.92 |
| Severe Patient 21 | 33.37 | 27.39 |
| Severe Patient 22 | 35.83 | 33.18 |
| Severe Patient 23 | 31.62 | 33.46 |
| Severe Patient 24 | 35.34 | 37.64 |
| Severe Patient 25 | 35.87 | 37.73 |
| Severe Patient 26 | 32.36 | 33.27 |
| Severe Patient 27 | 33.18 | 27.04 |
| Severe Patient 28 | 35.09 | 33.58 |
| Severe Patient 29 | 35.32 | 34.95 |
| Severe Patient 30 | 36.08 | 36.91 |
| Severe Patient 31 | 31.25 | 29.59 |
| Severe Patient 32 | 35.51 | 30.90 |
| Severe Patient 33 | 31.92 | 35.97 |
| Severe Patient 34 | 35.26 | 36.92 |
| Severe Patient 35 | 35.03 | 35.56 |
| Severe Patient 36 | 32.55 | 34.98 |
| Severe Patient 37 | 35.30 | 31.76 |
| Severe Patient 38 | 31.17 | 32.86 |
| Severe Patient 39 | 37.06 | 34.46 |
| Severe Patient 40 | 34.81 | 37.99 |

# Prediction cohort

## Severe/ Critically severe patients

| Patient 1 | | | |
|---|---|---|---|
| **$C_T$ value of viral genome** | **miR-nsp3-3p $C_T$ value** | | |
| **at admission** | **pre-severe stage** | **severe stage** | **recovery stage** |
| 30.24 | 40.13 | 33.85 | 35.75 |

| Patient 2 | | | |
|---|---|---|---|
| **$C_T$ value of viral genome** | **miR-nsp3-3p $C_T$ value** | | |
| **at admission** | **pre-severe stage** | **severe stage** | |
| 31.97 | 36.18 | 33.67 | 36.01 |

| Patient 3 | | | | | |
|---|---|---|---|---|---|
| **$C_T$ value of viral genome** | **miR-nsp3-3p $C_T$ value** | | | | |
| **at admission** | **pre-severe stage** | | **severe stage** | | |
| 32.45 | 36.62 | 34.12 | 33.00 | 32.6 | 32.46 |

| Patient 4 | | | | | |
|---|---|---|---|---|---|
| **$C_T$ value of viral genome** | **miR-nsp3-3p $C_T$ value** | | | | |
| **at admission** | **pre-severe stage** | **severe stage** | | | **recovery stage** |
| 32.02 | 35.29 | 32.74 | 31.36 | 35.26 | 38.59 |

| Patient 5 | | | | | |
|---|---|---|---|---|---|
| **$C_T$ value of viral genome** | **miR-nsp3-3p $C_T$ value** | | | | |
| **at admission** | **pre-severe stage** | **severe stage** | | **recovery stage** | |
| 31.12 | 35.68 | 34.39 | 35.21 | 36.89 | 36.55 |

| Patient 6 | | | |
|---|---|---|---|
| **$C_T$ value of viral genome** | **miR-nsp3-3p $C_T$ value** | | |
| **at admission** | **pre-severe stage** | **severe stage** | **recovery stage** |
| 27.90 | 36.03 | 34.94 | 38.29 |

| Patient 7 | | | |
|---|---|---|---|
| **$C_T$ value of viral genome** | **miR-nsp3-3p $C_T$ value** | | |
| **at admission** | **pre-severe stage** | **recovery stage** | |
| 31.97 | 34.68 | 37.83 | 43.41 |

| Patient 8 | | |
|---|---|---|
| **$C_T$ value of viral genome** | **miR-nsp3-3p $C_T$ value** | |
| **at admission** | **pre-severe stage** | **recovery stage** |
| 33.54 | 34.85 | 36.67 |

| Patient 9 | | | | | |
|---|---|---|---|---|---|
| **$C_T$ value of viral genome** | **miR-nsp3-3p $C_T$ value** | | | | |
| **at admission** | **pre-severe stage** | | **severe stage** | | |
| 31.08 | 34.76 | 32.23 | 32.58 | 33.26 | 33.88 |

## Mild/ Moderate patients

| Patient 10 | | |
|---|---|---|
| **$C_T$ value of viral genome at admission** | **miR-nsp3-3p $C_T$ value** | |
| 35.98 | 38.93 | 40.93 |

| Patient 11 | | |
|---|---|---|
| **$C_T$ value of viral genome at admission** | **miR-nsp3-3p $C_T$ value** | |
| 32.86 | 37.19 | 37.17 |

| Patient 12 | | | |
|---|---|---|---|
| **$C_T$ value of viral genome at admission** | **miR-nsp3-3p $C_T$ value** | | |
| 32.73 | 42.80 | 45.00 | 45.00 |

| Patient 13 | | | |
|---|---|---|---|
| **$C_T$ value of viral genome at admission** | **miR-nsp3-3p $C_T$ value** | | |
| 24.26 | 37.48 | 40.08 | 40.90 |

| Patient 14 | | |
|---|---|---|
| **$C_T$ value of viral genome at admission** | **miR-nsp3-3p $C_T$ value** | |
| 33.98 | 34.17 | 37.19 |

| Patient 15 |
|---|

| $C_T$ value of viral genome at admission | **miR-nsp3-3p $C_T$ value** | |
|---|---|---|
| 37.81 | 40.05 | 39.27 |

| Patient 16 | | | |
|---|---|---|---|
| **$C_T$ value of viral genome at admission** | **miR-nsp3-3p $C_T$ value** | | |
| 35.7 | 38.61 | 42.20 | 39.03 |

| Patient 17 | | |
|---|---|---|
| **$C_T$ value of viral genome at admission** | **miR-nsp3-3p $C_T$ value** | |
| 34.11 | 39.30 | 37.21 |

| Patient 18 | | |
|---|---|---|
| **$C_T$ value of viral genome at admission** | **miR-nsp3-3p $C_T$ value** | |
| 26.83 | 38.93 | 40.93 |

| Patient 19 | | | |
|---|---|---|---|
| **$C_T$ value of viral genome at admission** | **miR-nsp3-3p $C_T$ value** | | |
| 32.74 | 37.48 | 40.08 | 40.90 |

| Patient 20 | | | |
|---|---|---|---|
| **$C_T$ value of viral genome at admission** | **miR-nsp3-3p $C_T$ value** | | |
| 29.54 | 38.61 | 42.20 | 39.03 |
